# THE NEED FOR IMPROVED TACTILE ASSESSMENTS OF THE HAND IN OLDER ADULTS

**DOI:** 10.1093/geroni/igae098.2616

**Published:** 2024-12-31

**Authors:** Gül Kabil, Madeline Weber, Rachel Logue Cook, Cameron Haire, Daniel Duque Urrego, Stephen Cain, Paula Anne Newman-Casey, Susan Brown

**Affiliations:** University of Michigan, Ann Arbor, Michigan, United States; University of Michigan Medical School, Ann Arbor, Michigan, United States; University of Michigan, Ann Arbor, Michigan, United States; University of Michigan, Ann Arbor, Michigan, United States; West Virginia University, Morgantown, West Virginia, United States; West Virginia University, Morgantown, West Virginia, United States; University of Michigan, Ann Arbor, Michigan, United States; University of Michigan, Ann Arbor, Michigan, United States

## Abstract

Age-related sensorimotor declines of the hand significantly impact functional independence. Beyond muscle strength, tactile feedback provides valuable information regarding object characteristics that, in turn, can enhance manipulation skills. This is of particular interest in areas such as medication adherence - e.g. successful administration of eye drops or hearing aid adjustments. We investigated tactile acuity and dexterity in healthy young adults (23-35yo, n=23) and two groups of healthy older adults (65-76yo, n=24; 76-87yo, n=24). Participants completed assessments of strength (pinch, grip), dexterity (Grooved Pegboard Test, Arthritis Hand Function Test), tactile registration (Semmes-Weinstein Monofilament Test), and tactile pattern recognition using a custom-designed device. Both older groups performed significantly worse than young adults on all measures (p< 0.001). Dexterity, tactile identification accuracy, and tactile response times were significantly worse in the 76-87yo compared to the 65-76yo group (p=0.01). In contrast, strength and median monofilament scores did not differ between the two older adult groups. Across all older adults, tactile pattern recognition and dexterity were weakly correlated (p≤0.05). In the young adult group, only tactile accuracy and performance on the Arthritis Hand Function Test were correlated (p=0.04). These results suggest that clinical assessment of tactile acuity using monofilament testing may not capture declines in hand sensibility in older adults. More complex tactile assessments may be predictive of difficulties with object manipulation tasks that worsen with advanced age and negatively impact daily self-care activities.

